# How “Humane” Is Your Endpoint?—Refining the Science-Driven Approach for Termination of Animal Studies of Chronic Infection

**DOI:** 10.1371/journal.ppat.1002399

**Published:** 2012-01-19

**Authors:** Nuno H. Franco, Margarida Correia-Neves, I. Anna S. Olsson

**Affiliations:** 1 IBMC - Institute for Molecular and Cell Biology (Laboratory Animal Science Group), University of Porto, Porto, Portugal; 2 Life and Health Sciences Research Institute (ICVS), School of Health Sciences, University of Minho, Braga, Portugal; 3 ICVS/3B’s - PT Government Associate Laboratory, Braga/Guimarães, Portugal; The Fox Chase Cancer Center, United States of America

Public concern on issues such as animal welfare or the scientific validity and clinical value of animal research is growing, resulting in increasing regulatory demands for animal research. Abiding to the most stringent animal welfare standards, while having scientific objectives as the main priority, is often challenging. To do so, endpoints of studies involving severe, progressive diseases need to be established considering how early in the disease process the scientific objectives can be achieved. We present here experimental studies of tuberculosis (TB) in mice as a case study for an analysis of present practice and a discussion of how more refined science-based endpoints can be developed. A considerable proportion of studies in this field involve lethal stages, and the establishment of earlier, reliable indicators of disease severity will have a significant impact on animal welfare. While there is an increasing interest from scientists and industry in moving research in this direction, this is still far from being reflected in actual practice. We argue that a major limiting factor is the absence of data on biomarkers that can be used as indicators of disease severity. We discuss the possibility of complementing the widely used weight loss with other relevant biomarkers and the need for validation of these parameters as endpoints. Promotion of ethical guidelines needs to be coupled with systematic research in order to develop humane endpoints beyond the present euthanasia of moribund animals. Such research, as we propose here for chronic infection, can show the way for the development and promotion of welfare policies in other fields of research.

Research on chronic infection relies heavily on the use of animals, as only the integral animal body can model the full aspect of an infection. That animals are generally made to develop a disease in infection studies exacerbates the tension between human benefit and animal well-being, which characterizes all biomedical research with animals. Scientists typically justify animal research with reference to potential human benefits, but if accepting the assumption that human benefits can offset animal suffering, it still needs to be argued that the same benefits could not be achieved with less negative effects on animal welfare. Reducing the animal welfare problems associated with research (“refinement” [1]) is therefore crucial in order to render animal-based research less of an ethical problem and to assure public trust in research.

Studies that are designed to measure time of death or survival percentages present a particularly challenging situation in which at least some of the animals are made to die from the disease. These studies are frequent in experimental research on severe infections. The scientific community, industry, and regulatory authorities have responded to the ethical concerns over studies in which animals die from severe disease by developing new policies and guidelines for the implementation of humane endpoints as a key refinement measure (e.g., [2]–[4]). The most widely used definition considers a humane endpoint to be the earliest indicator in an animal experiment of severe pain, severe distress, suffering, or impending death [5], underlining that ideally such indicators should be identified before the onset of the most severe effects.

Euthanizing animals, rather than awaiting their “spontaneous” death, is important to avoid unnecessary suffering in studies in which data on survival is thought to be required for scientific or legal reasons. However, several questions remain open regarding how humane endpoints are to be applied to address real animal welfare problems. We used TB experiments in mice as a case study to highlight the potential to establish biomarkers of disease progress that can replace survival time as a measure of disease severity.

## Humane Endpoints Applied to Murine TB Experiments—State of the Arts

To illustrate the state of the art in the implementation of humane endpoints, we present data from a systematic review of articles published on murine TB in 2009. Papers were selected by performing an advanced search on the ISI Web of Science database (search performed in June 2011, using the previous 4.1 version, accessible at http://webofknowledgev4.com) with the terms “TS = ((mouse OR mice) SAME tuberculosis) AND PY = (2009)” and refined to exclude reviews, proceedings, articles in languages other than English, in vitro studies, and studies on other animal species and/or with other infectious agents than *Mycobacterium tuberculosis*, which resulted in a total of 80 papers reviewed. The severity of disease of the animals used in each study was analyzed in terms of the disease stage animals were allowed to reach, the carrying out of invasive procedures and the implementation of relevant refinement measures, and humane endpoints in particular. All studies in which animals were allowed to die spontaneously or to reach a moribund state were considered among the highest level of severity. Also, depending on whether studies were terminated before animals reached advanced stages of disease (which would rapidly progress towards spontaneous death if no other endpoints were applied) or not, these were classified as “lethal” or “non-lethal”, respectively. Lethal studies comprised those conducted in very susceptible strains as well as in more resistant mouse strains in which disease was allowed to progress to very advanced stages. Nearly half (47%) of the studies fit the lethal category, of which 66% were classified within the highest severity level. Although 26% of lethal studies explicitly reported having applied humane endpoints to avoid spontaneous death, in most of these studies (eight out of ten) humane endpoints were applied when animals were moribund and severely cachectic or when other animals in the same group began to die. Thus, most such endpoints consisted of merely replacing spontaneous death with euthanasia of moribund animals and may therefore fail to have a significant impact on the degree of poor animal well-being. Of the 25 studies considered within the highest severity, 24 had reported some sort of regulatory compliance, of which 17 explicitly stated that the study had been ethically approved.

## Are Near-Death Endpoints Really Humane?

The attention to humane endpoints is reflected in an increase in their implementation. This is clearly visible when reviewing research on murine TB: while the use of humane endpoints is reported in 26% of the terminal infection studies published in 2009, the same figure for 1999 is 15% (three of 20 papers reporting studies on lethal infections). However, a careful analysis reveals a significant limitation of humane endpoints as they are often presently applied. In fact, from both scientific and ethical perspectives, it is questionable if euthanasia of moribund animals is the most effective measure to prevent excessive animal suffering. It is admittedly difficult to estimate how much awareness of aversive sensations an individual retains in such a debilitated, unresponsive, and seemingly comatose state as the moribund animal is in [6]. But considering that the moribund state constitutes a terminal stage of a progressively distressing disease, the main animal welfare problem consists in the unrelieved suffering that precedes it. Furthermore, there is also the risk that researchers (as well as journal editors and animal ethics / animal care and use committees) may see euthanasia of moribund animals as a sufficient measure to guarantee the “ethicality” of their studies, thus hindering the pursuit for more welfare-relevant endpoints. An additional scientific concern pertains to the definition of the “moribund state” in itself; it is seldom defined in the material and methods section of the published articles. Therefore, it is unclear which clinical signs are used as indicators of imminent or pending death, and how soon before the estimated time of death they are detectable.

## The Potential for Scientific Refinement of Endpoints in Studies on Chronic Infection—Murine TB as a Case Study

We focus here on mice models for TB as a case study to discuss the potential for refining endpoints from both animal welfare and scientific viewpoints. TB is representative of chronic infection research in which, typically, adult animals are studied in experiments that last several weeks. The long duration means a greater potential**—**as well as a stronger animal welfare reason**—**for developing more refined early endpoints than in infections of a more acute nature. Mice experimentally infected with *M. tuberculosis* develop TB as a progressive disease. Although mouse strains vary in their susceptibility to infection [7], all mice eventually succumb to experimental TB [8], [9] and die before their natural average life-span if no measures are undertaken to treat them. Even the more TB-resistant C57BL/6 mice have a median survival time of fewer than 300 days [8], while uninfected animals live over 800 days [10], irrespective of the number of viable bacteria with which they are infected.

Time to death, when animals are found dead in the cage, is a frequently used outcome measure in experimental TB studies. Of studies published in 2009, 31% (25 out of 80) used death (18 out of 25) or the moribund stage (7 out of 25) as endpoints. Of notice and certainly as a consequence of awareness of animal welfare issues, an increasing number of studies are defining death as the time point when moribund animals are euthanized, rather than the time when they are found dead. This approach improves scientific accuracy both in that the exact time of death is known and in that biological samples can be collected immediately post-mortem. The informative value of time to death for understanding disease physiopathology is, however, limited by the fact that the actual cause of death may vary between animals: mice infected with *M. tuberculosis* may die from different lung pathologies in response to experimental infection [8], [11]–[13], as well as from causes only indirectly related to infection such as sepsis [14], hunger, or dehydration [15].

While euthanasia of moribund animals is an improvement compared to using death as an endpoint, as we have argued above, it is still a rather inefficient measure to safeguard animal welfare. Nevertheless, in the absence of validated predictors of death, this may presently be the best researchers can do if they need to establish whether animals reached a state from which they will deteriorate and die or might still recover. Identification of the turning point towards terminal disease, with greater understanding of the underlying host and pathogen factors leading to this stage, may provide endpoint measures that reliably reflect disease progression. Research on early predictors of a terminal disease stage (sometimes termed “surrogate endpoints” [16]), at times in which scientifically relevant data can be obtained before animals reach pronounced levels of distress, should therefore be encouraged.

Humane endpoint protocols are usually based on a combination of clinical signs; however, objectively and numerically measurable parameters are sometimes included [17]. Amidst these, percentage of body weight loss is the most commonly used as a cut-off parameter for euthanasia in experimental TB studies in mice, since it can be used as an objective measure of infection-related morbidity in this animal model (e.g., [18]). While non-infected mice tend to maintain or gain body weight, infected and untreated mice start to lose weight on account of active disease, in an irreversible and fairly linear manner [19], [20]–[23]. A consistent correlation between weight loss and survival has been reported specifically for backcrossed animals derived from A/Sn and I/St mice [23]–[25], which led Nikonenko and coworkers [25] to propose that drug efficacy can be evaluated through the systematic assessment of body weight changes in rapidly progressive TB in C3H mice, an immunocompetent but TB-susceptible strain.

Although this parameter is in itself quantitative and objectively measurable, the definition of the upper boundary values for euthanasia is potentially subjective, unless previously validated against data on disease progress and survival. Threshold weight losses vary considerably across studies, ranging from 15% of pre-infection weight [26] or of the average weight of control mice [27] to more severe 20% [28], 25% [29], or even 30% weight loss [30], with no scientific justification for choosing different cut-off points in different studies.

Systematic studies, in which data on biomarkers are collected repeatedly as the disease progresses and biomarker data is correlated with survival, are essential to develop new potential predictors of death/survival. Such studies need to be carried out specifically for distinct combinations of mouse and bacterial strains, with the aim of detecting selected biomarkers that indicate the point of no return after which animals do not recover. In addition to body weight, non-transient hypothermia has been suggested as a potentially useful predictor of death in some models of infection [31]–[36]. Hypothermia typically settles when disease reaches a near fatal stage and body temperature (which can be measured without handling stress by either infrared thermometers [35] or telemetric monitoring [36]) may constitute an important refinement of current moribund endpoints, particularly if scored with other clinical parameters [16]. A non-invasive method for the assessment of blood oxygen saturation in a murine model of viral pulmonary disease has also been shown to be a more reliable indicator of lung pathology and predictor of disease outcome than body weight loss [37]. This indicator of lung function has the potential to be used as a measure of morbidity in other pulmonary diseases if this can be validated in further research. Animals dying from distinct prolonged chronic infections have been shown to present other pathophysiological alterations that could be carefully studied as potential blood biomarkers of approaching death. These include anaemia and/or erythropoiesis and the metabolic perturbations in the glycolytic enzyme pathway with consequent altered blood glucose homeostasis, parameters that might be easily analyzed in blood samples and have been shown to correlate with the degree of disease severity [38], [39]. Biomarkers related to the immune response to infection are also described as potential indicators, reflecting the animals’ capacity to control the infection. In addition to the most widely studied cytokines associated with susceptibility/resistance to TB, like IFN-γ, TNF, IL-1, and IL-10 analyzed individually or in combinations [40]–[42], others have been suggested. A set of transcriptional biomarkers was recently shown to serve as diagnostic and prognostic tools, by the identification of a TB-specific 86-gene transcriptome signature in whole-blood samples, with the transcriptional signature being shown to correlate with extent of disease in patients with active TB and to also reflect changes at the site of disease [43]. However, while potentially clinically interesting, its measurement might be cumbersome unless a smaller number of transcripts proves to be still valid as molecular signature.

## Meeting Ethical Standards by a Science-Based Approach to Endpoints

It is praiseworthy that researchers, institutions, the public, and the authorities are all more aware of the need to establish ethical guidelines and rules in animal research to minimize distress. With that purpose, euthanasia is nowadays commonly established in most animal facilities when animals reach distress levels considered to be beyond an “acceptable” threshold [44]. There are, however, several problems with such a practice being imposed on research from external entities. First, the level found acceptable is bound to be somewhat arbitrary, depending on factors such as the research institution guidelines and the opinions of the appointed veterinarian, animal welfare officer, and members of the ethics committee. Second, from a scientific viewpoint, it is crucial to make sure that experimental objectives are not compromised by loss of experimental data due to unexpected deaths or compulsory euthanasia. Therefore, the establishment of earlier endpoints should ideally be centered on the earliest time point allowing the collection of adequate scientific information and valuable biological material, rather than on welfare-centered criteria.

As discussed above, in many cases additional research will be necessary to establish the correspondence of early clinical signs and final disease outcome. This calls for a new type of refinement research, in which measures to reduce animal welfare problems are developed specifically for the various fields of research and in close collaboration between laboratory animal specialists and researchers using animal models. Some such initiatives have emerged on the national level (e.g., National Centre for the 3Rs in the United Kingdom), but major international funding is unfortunately still limited to either the actual biomedical research itself or alternative methods to replace animal experiments, thus excluding studies on how to refine actual animal use.

Surrogate endpoints in the true sense constitute a crucial refinement of studies where a reliable measure of lethality is assumed to be necessary, in particular when these studies are long-term. We emphasize, however, the collection of informative data in less severe phases as the ideal approach for basic and applied research, allowing for a science-based, rather than welfare-centered justification for the termination of such experiments.

With respect to interventional euthanasia, although presently necessary to avoid further suffering of animals reaching unacceptably severe pain or other distress, it must be emphasized that this intervention ought to be more of an exception than the rule, and that studies should be planned so that such interventions are unnecessary.

## Conclusion

Animal research is valuable for identifying mechanisms of disease and novel therapies. The increased awareness of the ethical issues pertaining to animal experimentation requires that relevant scientific information and biological materials are obtained as early as possible, avoiding that animals have to reach severe stages of disease. In research on progressive diseases, it is pertinent to identify early surrogate endpoints of death as well as reliable biomarkers of disease progression. Changes in body weight and temperature, along with biomarkers easily measured in blood samples, should be carefully investigated as potential early predictors of death/survival. A science-driven approach for the termination of animal studies may not only prevent unnecessary and avoidable suffering, but also contribute to optimizing financial and human resources, enhancing the scientific output and speeding up the scientific process. The real humane endpoint challenge is to extend this approach into situations where presently the scientific outcome measure requires that animals reach more severe stages. While acknowledging current efforts by funding bodies and charities, regulators, industry, and academia in promoting and developing new standards for animal welfare, we stress that further collaborative research as well as funding for such research is essential to achieve more meaningful refinement of studies of chronic infections.

### Protein Accession Numbers/IDs

- Ifng interferon gamma [*Mus musculus*]; Gene ID: 15978; Protein ID: NP_032363
- Tnf tumor necrosis factor [*Mus musculus*]; Gene ID: 21926; Protein ID: NP_038721
- Il1b interleukin 1 beta [*Mus musculus*]; Gene ID: 16176; Protein ID: NP_032387
- Il10 interleukin 10 [*Mus musculus*]; Gene ID: 16153; Protein ID: NP_034678
